# Chasing the precursor of functional hematopoietic stem cells at the single cell levels in mouse embryos

**DOI:** 10.1186/s13045-016-0289-7

**Published:** 2016-07-22

**Authors:** Xiaochen Wang, Yuemin Gong, Hideo Ema

**Affiliations:** State Key Laboratory of Experimental Hematology, Institute of Hematology and Blood Diseases Hospital, Chinese Academy of Medical Sciences and Peking Union Medical College, Tianjin, 300020 China

**Keywords:** HSC, Pre-HSC, mTOR, Cell-cycle

## Abstract

**Background:**

Adult hematopoietic stem cells (HSCs), the ideal system for regenerative research, were isolated at single cell levels decades ago, whereas studies on embryonic HSCs are much more difficult.

**Methods:**

Zhou et al identified a new pre-HSC cell surface marker, CD201, by which they isolated pre-HSCs at single cell levels for further analyses.

**Results:**

The novel expression pattern of HSC development is revealed, including the fundamental role of mammalian targets of rapamycin (mTOR) signaling pathway in HSCs emergence, and the repopulation potential of S/G2/M phase pre-HSCs.

**Conclusions:**

Deeper understandings of the cellular origin and developmental regulatory network of HSCs are essential to develop new strategies of generating HSCs in vitro for clinical application.

Hematopoietic stem cells (HSCs) give rise to the entire blood cellular components. Due to the scarcity of HSCs (only one in every 10,000 to 15,000 bone marrow cells), only a limited number of the patients could have the chance to benefit from HSC transplantation, which is considered the most effective way to cure malignant hematologic diseases and many other genetic diseases. The supply of HSCs severely hinders broader applications of HSC in regenerative medicine. In 2006, Yamanaka invented the technology of reprogramming the somatic cells into the induced pluripotent stem (iPS) cells, which fundamentally altered the traditional theory that cell identity is fixed once terminally differentiated [1, 2]. The application of cell identity switches provides a potential strategy in generating enough quantity of HSCs for clinical application. However, to date, induction of HSCs from pluripotent stem cells, including embryonic stem cells and iPS cells, has been very inefficient, and thus has not been applied in the clinic. One of the obstacles is the elusiveness of the origin of HSCs. To generate functional HSCs requires capturing the precise stem cell state, which ensures retaining their proliferation and self-renewal activity after engraftment. Deeper understandings of HSC-specific cell identity as well as their regulatory pathways still need to be made.

During mammalian embryonic development, at least two waves of hematopoiesis take place, primitive hematopoiesis that generates mostly myeloid cells and erythrocytes, and definitive hematopoiesis that produces HSCs with long-term reconstitution and multi-lineage potential. Definitive HSCs are believed to be derived from hemogenic endothelium cells (EC). The earliest HSCs can be detected at embryonic day (E) 10.5 in aorta-gonad-mesonephros (AGM) region, when the number of definitive HSCs is less than one per embryo [3]. Around E11, two types of immature HSCs, CD45^−^ pre-HSCs (T1 pre-HSCs) and later CD45^+^ pre-HSCs (T2 pre-HSCs), were identified to be capable of developing into long-term repopulating definitive HSCs [4]. Then, the CD45^+^ HSCs start to migrate into fetal liver (FL) at E11.5, where the HSCs experience massive expansion for 4–5 days, and then be released into circulating blood and finally settle in the future adult bone marrow and spleen. To specify gene expression patterns of HSC precursors and HSCs at different stages [5] is fundamental for elucidating HSCs cell identity transitions.

Due to the small number, limited time span, and rapid change of surface markers of HSCs during the early stage of embryonic development, it is of great difficulty to clarify the mechanisms in gene expression levels. Therefore, single cell tracing would be an ideal approach, which has been used to achieve the temporal regulatory gene expression profiling of hematopoietic differentiation for adult HSCs [6, 7]. Recently, Zhou et al captured the pre-HSCs at the single cell levels for the first time, and performed the single cell RNA-seq analyses, in which they identified new markers for pre-HSC isolation, revealed indispensable role of activation of mammalian targets of rapamycin (mTOR)/Rictor pathway in HSC generation, and demonstrated unique cell-cycle status of pre-HSCs, thus to advance the knowledge of complex regulation of HSCs during development [8] (Fig. 1).

**Fig. 1:**
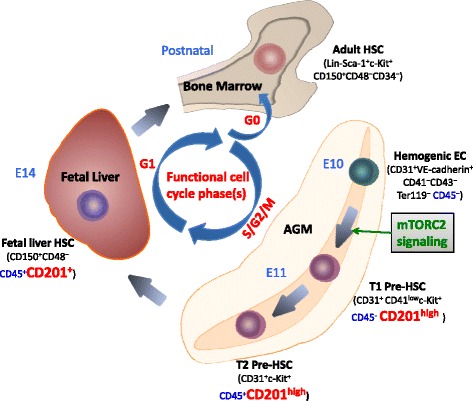
New findings on precursors of HSCs. Hematopoietic stem cells (HSCs) are believed to develop from hemogenic endothelium (EC) cells. The earliest HSCs that are capable of reconstitution potential emerge at embryonic day (E) 10.5 in aorta-gonad-mesonephros (AGM) region, then later migrate to fetal liver, and finally settle in the future adult bone marrow and spleen. The identification of cell surface markers of different stage HSCs facilitate for efficient enrichment of HSCs, thus to elucidate HSC development transcriptional machinery. Recently, CD201 (endothelial protein C receptor, EPCR) was identified as a pre-hematopoietic stem cells (pre-HSCs) surface marker for the first time. By isolation and analyses of pre-HSCs at single cell levels, unique cell-cycle features of pre-HSCs were discovered. Different from past studies that most of adult HSCs reside in G0 phase and none of the S/G2/M phase adult HSCs could successfully reconstitute, transplantation of S/G2/M phase pre-HSCs demonstrates most robust reconstitution capability, G0 phase pre-HSCs show moderate activity, and none of the G1 phase pre-HSCs is able to reconstitute. During the development of HSCs, activation of mammalian targets of rapamycin (mTOR) signaling pathway is fundamental for HSCs emergence

CD201 (also known as endothelial protein C receptor, EPCR), combined with SLAM markers has been used to define a rare population with HSC activity throughout lifetime [9]. Previous analyses on embryonic HSCs began from E14.5 fetal liver when HSCs are able to be transplanted directly without in vitro priming. Yet the specific markers to enrich HSC precursors from early embryonic days remain unexplored. In combination with other T1 pre-HSC markers (CD31^+^CD45^−^CD41^low^c-Kit^+^CD201^high^), Zhou et al isolated single T1 pre-HSC cells, which showed robust self-renewal capability in serial transplantations. Single cell RNA-seq analyses were performed using samples from five different stages of HSC precursors and HSCs (E11 ECs, E11 T1 pre-HSCs, E11 T2 pre-HSCs, E12 and E14 fetal liver HSCs), of which the result suggests that one subpopulation among T2 pre-HSCs (CD31^+^CD45^+^c-Kit^+^) showed similar gene signatures to T1 pre-HSCs with high expression of CD201. Single cell transplantation confirmed the enrichment of HSC potential cells in this CD201^high^ T2 subpopulation. Zhou et al’s work showed that CD201 is a potent pre-HSC enrichment marker for the first time. The discovery of new pre-HSC maker ensures single cell acquisition throughout the entire HSC development hierarchy, and provides reliable basis for further understanding of HSC development.

Single cell RNA-seq analyses also revealed new signaling pathways controlling HSC formation, among which mTOR signaling pathway stood out for its potential role in regulating HSC function. Noticing overt mTORC2 activation in T1 pre-HSCs, Zhou et al conducted function analysis using Rictor (a main component of the MTOR2 complex) conditional knock-out murine models. They specified that Rictor is required for HSC emergence from endothelium but not for later HSC maintenance and function, nor hematopoietic progenitor cells (HPCs) generation.

Another remarkable finding is the complete difference of cell-cycle status of pre-HSCs. After birth, most adult HSCs settle in the bone marrow, where they reside in quiescence and mainly remain in G0 phase [10, 11]. Different from adult HSCs, in which the S/G2/M phases HSCs were found inactive, S/G2/M phase pre-HSCs show most vigorous proliferation and reconstitution ability, G0 phase pre-HSCs show moderate repopulation potential, and none of them are in G1 phase. This novel observation still needs further investigation to clarify why functional HSCs at different stage diverse so tremendously in cell-cycle status.

The reconstitution potential of pre-HSCs that Zhou et al showed in their work may potentially provide an alternative strategy for regenerative approaches. HSCs are known to have multi-lineage hematopoietic differentiation potential and sustain self-renewal activity, and always are the dream target of induction from pluripotent stem cells. Now that more pre-HSC markers are identified, induction of pre-HSC, which shares abundant features with HSCs, might be of interests in regenerative medicine research and prospects for therapy.

More importantly, the single cell RNA-seq data of the five different stages of HSC precursors and HSCs provide the possibility of revealing the full spectrum of HSC development transcriptional machinery. Notably, one of the 98 pre-HSC signature genes, α-catulin, was reported to be pivotal for adult HSCs last year [12]. The identification of pre-HSC signature genes (including genes related to transcriptional activity, arterial endothelium, metabolism, movement, etc.) provide a great avenue to the understanding of HSC development, differentiation, and reprogramming.

Questions still remain like whether we could track the precursors of the pre-HSCs, i.e., *Flk-1*^*+*^ mesodermal cells. Only about one third of the pre-HSC single cells are capable of repopulation, does this HSC heterogeneity owe to different cell-cycle status or more specified development stages? Besides the HSC intrinsic regulation, does the embryonic microenvironment play a role in pre-HSC development? The most important issue that needs to be addressed in the next step is in vivo tracking of pre-HSCs in both directions (earlier embryonic stage and adult aged stage), particularly the role of embryonic pre-HSCs and HSCs in adult hematopoiesis. To generate functional HSCs from pluripotent stem cells for the need of regenerative medicine, we ought to know much more if not all components of the molecular network that governs HSC identity.

## Abbreviations

AGM, aorta-gonad-mesonephros; E, embryonic day; EC, endothelium cell; EPCR, endothelial protein C receptor; HPC, hematopoietic progenitor cell; HSC, hematopoietic stem cell; iPS cell, induced pluripotent stem cell; mTOR, mammalian targets of rapamycin; Pre-HSC, Pre-hematopoietic stem cell
